# Supplementary material for: A UMLS-based spell checker for natural language processing in vaccine safety
Source: BMC Med Inform Decis Mak. 2007 Feb 12;7:3. doi: 10.1186/1472-6947-7-3 (PMC1805499; doi:10.1186/1472-6947-7-3)
Supplement: Additional file 13 — RAP application directory. Sets up directory for RAP (RDF application for PHP) source code [file 1472-6947-7-3-S13.gz › rap/doc/phpdoc/__filesource/fsource_syntax__syntaxN3Parser.php.html]

xml version="1.0" encoding="iso-8859-1"?


File Source for N3Parser.php


# Source for file N3Parser.php

Documentation is available at N3Parser.php

```
1. <?php

3. // ----------------------------------------------------------------------------------
4. // Class: N3Parser
5. // ----------------------------------------------------------------------------------


11. /**
12. * PHP Notation3 Parser
13. *
14. * This parser can parse a subset of n3, reporting triples to a callback function
15. * or constructing a RAP Model ( http://www.wiwiss.fu-berlin.de/suhl/bizer/rdfapi )
16. *
17. * Supported N3 features:
18. * <ul>
19. *   <li>Standard things, repeated triples ( ; and , ), blank nodes using [ ], self-reference ('<>')</li>
20. *   <li>@prefix mappings</li>
21. *   <li>= maps to owl#sameAs</li>
22. *   <li>a maps to rdf-syntax-ns#type</li>
23. *   <li>Literal datytype- and xmlLanguageTag support
24. * </ul>
25. * Un-supported N3 Features include:
26. * <ul>
27. *   <li>Reification using { }</li>
28. *   <li>. and ^ operators for tree traversal</li>
29. *   <li>Any log operators, like log:forAll etc.</li>
30. * </ul>
31. *
32. * This parser is based on n3.py from Epp released 2nd March, 2002.
33. * by Sean B. Palmer
34. * ( http://infomesh.net/2002/eep/20020302-013802/n3.py )
35. *
36. * This parser is released under the GNU GPL license.
37. * ( http://www.gnu.org/licenses/gpl.txt )
38. *
39. * <b>History:</b>
40. * <ul>
41. * <LI>04-05-2005 toke() function improved by Hannes Gassert hannes.gassert@deri.org </LI>
42. * <LI>03-25-2005 N3 list processing added by Hannes Gassert hannes.gassert@deri.org</LI>
43. * <LI>12-06-2004 improved namespace handling added (tobias.gauss@web.de)</LI>
44. * <LI>08-10-2004 Function for converting strings to its unicode NFC form. Benjamin Nowack <bnowack@appmosphere.com></LI>
45. * <LI>10-05-2004 Fixed bug with trailing space on qnames and space before ] parsin bug
46. * <LI>11-27-2003 fixed problems with whithespaces at the end of bNodes</li>
47. * <LI>11-18-2003 Changed xml:language regex for supporting lang-tags like en-uk.</li>
48. * <li>11-07-2003 Added "setFixBnodes" function. Sets, if Bnodes should be renamed to the BNODE_PREFIX constant.</li>
49. * <li>10-27-2003 fixed problems in generateModel(), changed regEx for Literals.</li>
50. * <li>10-24-2003 Added support for Literals with rdf:DataType and xml:Language Tags. URI-Self-Reference with '<>' is supported.</li>
51. * <li>08-01-2003 Made compatible with new v6 MemModel.</li>
52. * <li>07-31-2003 Function generateModel() added.</li>
53. * <li>07-16-2003 Fixed bug with anon nodes alone on a line.</li>
54. * <li>06-08-2003 Initial version converted from n3.py.</li>
55. * </ul>
56. *
57. *
58. * @author Sean B. Palmer <sean@mysterylights.com>, Gunnar AA. Grimnes <ggrimnes@csd.abdn.ac.uk>, Daniel Westphal <mail@d-westphal.de>
59. * @version V0.9.1
60. * @package syntax
61. * @access public
62. ***/

64. class N3Parser extends Object {


67. /* ==================== Variables ==================== */

69. var $Tokens;
70. var $bNode;
71. var $RDF_NS, $DAML_NS, $OWL_NS;
72. var $debug;
73. var $parseError;
74. var $parsedNamespaces = array();

76. /* ==================== Public Methods ==================== */

78. /**
79. * Constructor
80. * @access public
81. ***/
82. function N3Parser() {
83. //Regular expressions:
84. $Name = '[A-Za-z0-9_@\.]+[^\.,;\[\] ]*';
85. $URI = '<[^> ]*>';
86. $bNode = '_:'.$Name;
87. $Univar = '\?'.$Name;
88. $QName = '(?:[A-Za-z][A-Za-z0-9_@\.]*)?:'.$Name;
89. $Literal = '"(\\\"|[^"])*"'; # '"(?:\\"|[^"])*"'
90. //   $Literal = '"[^"\\\\]*(?:\\.\\[^"\\]*)*"'; # '"(?:\\"|[^"])*"'
91. $LangTag = '@[A-Za-z\-]*[^ \^\.\;\,]';
92. $Datatype = '(\^\^)[^ ,\.;)]+';
93. $Datatype_URI = '(\^\^)'.$URI;
94. //     $LLiteral = '"""[^"\\\\]*(?:(?:.|"(?!""))[^"\\\\]*)*"""';
95. $LLiteral = '"""[^"\\\\]*(?:(?:\\\\.|"(?!""))[^"\\\\]*)*"""';
96. //          '"""[^"\\]*(?:(?:\\.|"(?!""))[^"\\]*)*"""'
97. $Comment = '# .*$';
98. $Prefix = '(?:[A-Za-z][A-Za-z0-9_]*)?:';
99. $PrefixDecl = '@prefix';
100. $WS = '[ \t]';
101. $this->RDF_NS = 'http://www.w3.org/1999/02/22-rdf-syntax-ns#'; # for 'a' keyword
102. $this->DAML_NS = 'http://www.daml.org/2001/03/daml+oil#'; # for '=' keyword
103. $this->OWL_NS = 'http://www.w3.org/2002/07/owl#';

105. //     $t = array( $LLiteral, $URI); //, $Literal, $PrefixDecl, $QName, $bNode, $Prefix,
106. //        $Univar, 'a', '{', '}', '\(', '\)', '\[', '\]', ',', ';', '\.', $WS, $Comment);
107. $t = array( $Datatype_URI,$Datatype,$LLiteral, $URI, $Literal, $PrefixDecl, $QName, $bNode, $Prefix, $Univar, 'a','=', '{', '}', '\(', '\)', '\[', '\]', ',', ';', '\.', $WS, $Comment,$LangTag);
108. $this->Tokens="/(".join($t,"|").")/m";

110. $this->bNode=0;
111. $this->debug=0;
112. $this->bNodeMap = array();
113. $this->FixBnodes = FIX_BLANKNODES;
114. $this->parseError=false;
115. }


118. /**
119. * Sets, if BlankNode labels should be replaced by the generic label from the constants.php file
120. * default is "false" -> the used label in n3 is parsed to the model
121. * @param boolean
122. * @access public
123. ***/
124. function setFixBnodes($set) {

126. if (($set===true) OR ($set===false)) $this->FixBnodes = $set;
127. }


130. /**
131. * This parses a N3 string and prints out the triples
132. * @param string $s
133. * @access public
134. ***/
135. function parse($s) {
136. //   """Get a string, tokenize, create list, convert to Eep store."""
137. $stat=$this->n3tolist($s);
138. foreach ( $stat as $t) {

140. if (count($t)>3) {
141. $object=$t[2];

143. for ($i = 3; $i < 5; $i++){
144. if ($t[$i][0]=='@')$object.=$t[$i];
145. if (substr($t[$i],0,2)=='^^')$object.=$t[$i];
146. };
147. } else {$object=$t[2];};

149. print '('.$t[0].', '.$t[1].', '.$object.")\n";

151. }
152. //   return [[eep.Article(t[0]), eep.Article(t[1]), eep.Article(t[2])]
153. //              for t in n3tolist(s)]
154. }


157. /**
158. * This parses a N3 string and calls func($subject, $predicate, $object) with each trioke
159. * @param string $s
160. * @param string $func
161. * @access public
162. ***/
163. function uparse($s,$func) {
164. //   """Get a string, tokenize, create list, convert to Eep store."""
165. $stat=$this->n3tolist($s);
166. foreach ( $stat as $t) {

168. if (count($t)>3) {
169. $object=$t[2];

171. for ($i = 3; $i < 5; $i++){
172. if ($t[$i][0]=='@')$object.=$t[$i];
173. if (substr($t[$i],0,2)=='^^')$object.=$t[$i];
174. };
175. } else {$object=$t[2];};
176. //    print "(".$t[0].", ".$t[1].", ".$t[2].")";

178. $func($t[0],$t[1],$object);
179. }
180. //   return [[eep.Article(t[0]), eep.Article(t[1]), eep.Article(t[2])]
181. //              for t in n3tolist(s)]
182. }


185. /**
186. * This parses a N3 string and returns a memmodel
187. * @param string $s
188. * @access public
189. * @return object Model
190. ***/

192. function parse2model($s,$model = false) {
193. if($model == false){
194. $m=new MemModel();
195. }else{
196. $m=$model;
197. }
198. //   """Get a string, tokenize, create list, convert to Eep store."""
199. $stat=$this->n3tolist($s);

201. foreach ( $stat as $t) {
202. $s=$this->toRDFNode($t[0],$t);
203. $p=$this->toRDFNode($t[1],$t);
204. $o=$this->toRDFNode($t[2],$t);

206. $new_statement= new Statement($s,$p,$o);

208. $m->add($new_statement);
209. //    print "(".$t[0].", ".$t[1].", ".$t[2].")";
210. }
211. //   return [[eep.Article(t[0]), eep.Article(t[1]), eep.Article(t[2])]
212. //              for t in n3tolist(s)]

214. $m->addParsedNamespaces($this->parsedNamespaces);
215. return $m;
216. }

218. /**
219. * Generate a new MemModel from an URI or file.
220. *
221. * @access    public
222. * @param $path
223. * @throws PhpError
224. * @return object MemModel
225. */
226. function & generateModel($path,$dummy=false,$model=false) {

228. $handle = fopen($path,'r') or die("N3 Parser: Could not open File: '$path' - Stopped parsing.");
229. $done=false;
230. $input="";
231. while(!$done)
232. {
233. $input .= fread( $handle, 512 );
234. $done = feof($handle);

236. };


239. fclose($handle);

241. return $this->parse2model($input,$model);
242. }


245. /* ==================== Private Methods from here ==================== */

247. //  General list processing functions


251. /**
252. * Returns FALSE if argument is a whitespace character
253. * @access private
254. * @param string $s
255. ***/
256. function isWS($s) {
257. return !preg_match('/^(#.*|\s*)$/', $s);
258. }


262. /**
263. * Returns true if the string is not a comment
264. * @access private
265. * @param string $s
266. * @returns boolean
267. ***/
268. function notComment($s) {
269. if ($s=="") return false;
270. $N3Comment = '^[ \t]*\#';

272. if (ereg($N3Comment,$s)) return false;
273. else return true;
274. }

276. /**
277. * Removes all whitespace tokens from list
278. * @access private
279. * @param array $list
280. ***/
281. function filterWs($list) {
282. //    var_dump($list);
283. //  """Filter whitespace from a list."""

285. return array_filter($list, array($this,"isWS"));
286. }

288. /**
289. * converts a string to its unicode NFC form (e.g. \uHHHH or \UHHHHHHHH).
290. *
291. * @param String $str
292. * @return String
293. * @access private
294. *
295. */
296. function str2unicode_nfc($str=""){
297. $result="";
298. /* try to detect encoding */
299. $tmp=str_replace("?", "", $str);
300. if(strpos(utf8_decode($tmp), "?")===false){
301. $str=utf8_decode($str);
302. }
303. for($i=0,$i_max=strlen($str);$i<$i_max;$i++){
304. $nr=0;/* unicode dec nr */
305. /* char */
306. $char=$str[$i];
307. /* utf8 binary */
308. $utf8_char=utf8_encode($char);
309. $bytes=strlen($utf8_char);
310. if($bytes==1){
311. /* 0####### (0-127) */
312. $nr=ord($utf8_char);
313. }
314. elseif($bytes==2){
315. /* 110##### 10###### = 192+x 128+x */
316. $nr=((ord($utf8_char[0])-192)*64) + (ord($utf8_char[1])-128);
317. }
318. elseif($bytes==3){
319. /* 1110#### 10###### 10###### = 224+x 128+x 128+x */
320. $nr=((ord($utf8_char[0])-224)*4096) + ((ord($utf8_char[1])-128)*64) + (ord($utf8_char[2])-128);
321. }
322. elseif($bytes==4){
323. /* 1111#### 10###### 10###### 10###### = 240+x 128+x 128+x 128+x */
324. $nr=((ord($utf8_char[0])-240)*262144) + ((ord($utf8_char[1])-128)*4096) + ((ord($utf8_char[2])-128)*64) + (ord($utf8_char[3])-128);
325. }
326. /* result (see http://www.w3.org/TR/rdf-testcases/#ntrip_strings) */
327. if($nr<9){/* #x0-#x8 (0-8) */
328. $result.="\\u".sprintf("%04X",$nr);
329. }
330. elseif($nr==9){/* #x9 (9) */
331. $result.='\t';
332. }
333. elseif($nr==10){/* #xA (10) */
334. $result.='\n';
335. }
336. elseif($nr<13){/* #xB-#xC (11-12) */
337. $result.="\\u".sprintf("%04X",$nr);
338. }
339. elseif($nr==13){/* #xD (13) */
340. $result.='\t';
341. }
342. elseif($nr<32){/* #xE-#x1F (14-31) */
343. $result.="\\u".sprintf("%04X",$nr);
344. }
345. elseif($nr<34){/* #x20-#x21 (32-33) */
346. $result.=$char;
347. }
348. elseif($nr==34){/* #x22 (34) */
349. $result.='\"';
350. }
351. elseif($nr<92){/* #x23-#x5B (35-91) */
352. $result.=$char;
353. }
354. elseif($nr==92){/* #x5C (92) */
355. $result.='\\';
356. }
357. elseif($nr<127){/* #x5D-#x7E (93-126) */
358. $result.=$char;
359. }
360. elseif($nr<65536){/* #x7F-#xFFFF (128-65535) */
361. $result.="\\u".sprintf("%04X",$nr);
362. }
363. elseif($nr<1114112){/* #x10000-#x10FFFF (65536-1114111) */
364. $result.="\\U".sprintf("%08X",$nr);
365. }
366. else{
367. /* other chars are not defined => ignore */
368. }
369. }
370. return $result;
371. }


375. /**
376. * Gets a slice of an array.
377. * Returns the wanted slice, as well as the remainder of the array.
378. * e.g. getSpan(['p', 'q', 'r'], 1, 2) gives (['q'], ['p', 'r'])
379. * @return array
380. * @access private
381. * @param array $list
382. * @param integer $start
383. * @param integer $end
384. ***/
385. function getSpan($list, $start, $end) {

387. $pre=array_slice($list, 0, $start);
388. $post=array_slice($list, $end);

390. return array(array_slice($list, $start,$end-$start),$this->array_concat($pre,$post));
391. }


394. /**
395. * Concatenates two arrays
396. * @param array $a
397. * @param array $b
398. * @returns array
399. * @access private
400. ***/
401. function array_concat($a, $b) {
402. array_splice($a,count($a),0,$b);
403. return $a;
404. }

406. /**
407. * Returns an array with all indexes where item appears in list
408. * @param array $list
409. * @param string $item
410. * @returns array
411. * @access private
412. ***/
413. function posns($list, $item) {
414. $res=array();
415. $i=0;
416. foreach ( $list as $k=>$v) {
417. if ($v === $item ) $res[]=$i;
418. $i++;
419. }
420. $res[]=$i;
421. return $res;
422. }


425. /* More N3 specific functions */

427. /**
428. * Returns a list of tokens
429. * @param string $s
430. * @returns array
431. * @access private
432. ***/
433. function toke($s) {

435. //    print "$s\n";
436. //   """Notation3 tokenizer. Takes in a string, returns a raw token list."""
437. if (strlen($s) == 0) die('Document has no content!');

439. $s=str_replace("\r\n","\n",$s);
440. $s=str_replace("\r","\n",$s);


443. //$lines=explode("\n",$s);

445. //$reallines=array_filter($lines, array($this, "notComment"));
446. //    print "LINES: ".join($reallines, " ")." :LINES\n";
447. //array_walk($reallines, array($this, "trimLine"));
448. //$res=array();

450. //    foreach ($reallines as $l) {
451. //preg_match_all($this->Tokens, $l, $newres);
452. //$res=$this->array_concat($res,$newres[0]);
453. //}

455. $res=array();
456. preg_match_all($this->Tokens, $s, $newres);

458. $res=$this->array_concat($res, array_map('trim', $newres[0]));

460. return $res;
461. }

463. /**
464. * Returns a list with the elements between start and end as one quoted string
465. * e.g. listify(["a","b","c","d"],1,2) => ["a","b c", "d"]
466. * @param array $list
467. * @param integer $start
468. * @param integer $end
469. * @returns array
470. * @access private
471. ***/
472. function listify($list, $start, $end) {

474. //Re-form a list, merge elements start->end into one quoted element
475. //Start and end are offsets...

477. $l=$end-$start;

479. $s=array_slice($list, 0, $start);
480. $m=array_slice($list, $start,$l);
481. $e=array_slice($list, $end);

483. //  array_push($s,"\"".join($m," ")."\"");
484. array_push($s,$m);

486. return $this->array_concat($s,$e);
487. }

489. /**
490. * Returns an array with prefixes=>namespace mappings
491. * @param array $list
492. * @access private
493. * @returns array
494. ***/
495. function getPrefixes($list) {

497. $prefixes=array();
498. $ns=1;
499. $name=2;
500. foreach ($list as $l) {
501. if ($l=='@prefix') {
502. //   while '@prefix' in list {

504. $pos=current($list);
505. //pos = list.index('@prefix')
506. $r = $this->getSpan($list, $pos, ($pos+4)); # processes the prefix tokens
507. $binding=$r[0];
508. $list=$r[1];
509. $prefixes[$binding[$ns]] = substr($binding[$name],1,-1);
510. $this->parsedNamespaces[substr($binding[$name],1,-1)] = substr($binding[$ns],0,-1);
511. }
512. }

514. if (count($prefixes)<1) $list= array_slice($list,0);

516. return array($prefixes, $list);
517. }

519. /**
520. * Callback function for replacing "a" elements with the right RDF uri.
521. * @param string $l
522. * @access private
523. ***/
524. function replace_a_type(&$l,$p) {
525. if ($l=='a') $l='<'.$this->RDF_NS.'type>';
526. }

528. /**
529. * Callback function for replacing "=" elements with the right DAML+OIL uri.
530. * @param string $l
531. * @access private
532. ***/
533. function replace_equal(&$l,$p) {
534. if ($l=='=') $l='<'.$this->OWL_NS.'sameAs>';
535. }

537. /**
538. * Callback function for replacing "this" elements with the right RDF uri.
539. * @param string $l
540. * @access private
541. ***/
542. function replace_this($l,$p) {
543. if ($l=='this') $l='<urn:urn-n:this>';
544. }

546. /**
547. * Applies stuff :)
548. * Expands namespace prefixes etc.
549. * @param array $prefixes
550. * @param array $list
551. * @returns $list
552. * @access private
553. ***/
554. function applyStuff($prefixes, $list) {

556. array_walk($list, array($this, 'replace_a_type'));
557. array_walk($list, array($this, 'replace_equal'));
558. array_walk($list, array($this, 'replace_this'));

560. for ($i=0;$i<count($list);$i++) {
561. //   for i in range(len(list)) {
562. //      if (!strstr('<_"?.;,{}[]()',$list[$i]{0})) {


565. // if a <> resource occours, change it to the parsed filename or local URI + timestamp

567. if ($list[$i]=='<>') {
568. if (!isset($path)) {
569. if (!isset($_SERVER['SERVER_ADDR'])) $_SERVER['SERVER_ADDR']='localhost';
570. if (!isset($_SERVER['REQUEST_URI'])) $_SERVER['REQUEST_URI']='/rdfapi-php';
571. $list[$i]='<http://'.$_SERVER['SERVER_ADDR'].$_SERVER['REQUEST_URI'].'#generate_timestamp_'.time().'>';

573. }else {$list[$i]='<'.$path.'>';};
574. };


577. if ((!strstr('<_"?.;,{}[]()@',$list[$i]{0}))AND (substr($list[$i],0,3)!='^^<')) {
578. $_r= explode(":",$list[$i]);


584. $ns=$_r[0].':';
585. $name=$_r[1];

587. if (isset($prefixes[$ns])) $list[$i] = '<'.$prefixes[$ns].$name.'>';
588. else if (isset($prefixes[substr($ns,2)])) $list[$i] = '^^'.$prefixes[substr($ns,2)].$name.'';
589. else {
590. #die('Prefix not declared:'.$ns);
591. $this->parseError=true;
592. trigger_error('Prefix not declared: '.$ns, E_USER_ERROR);
593. break;


596. }

598. } else {
599. if ($list[$i]{0} == '"') {  // Congratulations - it's a literal!
600. if (substr($list[$i],0,3) == '"""') {
601. if (substr($list[$i],-3,3) == '"""') { // A big literal...
602. $lit = substr($list[$i],3,-3);
603. //          print "++$lit++";
604. $lit=str_replace('\n', '\\n',$lit);

606. $lit=ereg_replace("[^\\]\"", "\\\"", $lit);

608. $list[$i] = '"'.$lit.'"';
609. }
610. else { die ('Incorrect string formatting: '.substr($list[$i],-3,3)); }
611. } else {
612. if (strstr($list[$i],"\n")) die('Newline in literal: '+$list[$i]);
613. }
614. }
615. }

617. if (substr($list[$i],0,2)=='^^') {
618. if ($list[$i][2]!='<'){$list[$i]='^^<'.substr($list[$i],2).'>';};
619. };


622. }


625. return $list;
626. }

628. /**
629. * Returns an array of triples extracted from the list of n3 tokens
630. * @param array $list
631. * @returns array
632. * @access private
633. ***/
634. function getStatements($list) {


637. $statements = array();

639. while (in_array('.', $list)) {
640. //  for($i=0;$i<count($list); $i++) {
641. //    if ($list[$i]==".") {
642. //   while '.' in list {
643. $pos=array_search('.',$list);

645. $r=$this->getSpan($list, 0, $pos+1);

647. $statement=$r[0];
648. $list = $r[1];

650. array_pop($statement);
651. $statements[]=$statement;
652. }

654. return $statements;
655. }

657. /**
658. * Gets a list of triples with same subject
659. * e.g. :Gunnar :firstname "Gunnar" ; :lastname "Grimnes.
660. * @param array $list
661. * @returns array
662. * @acces private
663. ***/
664. function getPovs($list) {
665. $povs = array();
666. while (in_array(';', $list)) {
667. $r=$this->posns($list,';');
668. $pos=array_slice($r,0,2);
669. $r = $this->getSpan($list, $pos[0], $pos[1]);
670. $pov=$r[0];
671. $list=$r[1];

673. $povs[]=array_slice($pov,1);
674. }

676. return array($list, $povs);
677. }

679. /**
680. * Gets a list of triples with same predicate
681. * e.g. :Gunnar :likes "Cheese", "Wine".
682. * @access private
683. * @param array $list
684. * @returns array
685. ***/
686. function getObjs($list) {


689. $objs = array();
690. while (in_array(",",$list)) {
691. $pos=array_search(",",$list);
692. //  for($i=0;$i<count($list); $i++) {
693. //    if ($list[$i]==",") {
694. //   while ',' in list {


697. $get_array_fields=2;
698. if (isset ($list[$pos+2])) {
699. if (@$list[$pos+2][0]=='@') $get_array_fields++;
700. if (@$list[$pos+2][0]=='^') $get_array_fields++;
701. };
702. if (isset ($list[$pos+3])) { if (@$list[$pos+3][0]=='^') $get_array_fields++;};


705. $r=$this->getSpan($list, $pos, ($pos+$get_array_fields));

707. $obj=$r[0];
708. if (!isset($obj[2])) $obj[2]=' ';
709. if (!isset($obj[3])) $obj[3]=' ';

711. $list=$r[1];


714. $objs[]=$obj;


717. }

719. return array($list, $objs);
720. }

722. /**
723. * Does the real work, returns a list of subject, predicate, object triples.
724. * @param array $list
725. * @returns array
726. * @access private
727. ***/
728. function statementize($list) {

730. if (count($list) == 1 && preg_match("/_".BNODE_PREFIX."[0-9]+_/",$list[0])) {
731. if ($this->debug) print "Ignored bNode exists statement. $list\n";
732. return array();
733. }


737. if (count($list) == 3) return array($list);
738. if (count($list) < 3) die("Error: statement too short!");

740. //Get all ;
741. $r=$this->getPovs($list);
742. $spo=$r[0];
743. $po=$r[1];
744. $all=array();


748. //      (spo, po), all = getPovs(list), []
749. $subject = $spo[0];
750. foreach ($po as $pop) {
751. //  for pop in po {
752. $r=$this->getObjs($pop);

754. $myPo=$r[0];
755. $obj=$r[1];
756. //myPo, obj = getObjs(pop)

758. if (!isset($myPo[2])) $myPo[2]=' ';
759. if (!isset($myPo[3])) $myPo[3]=' ';


762. $predicate = $myPo[0];
763. $all[]=array($subject,$predicate,$myPo[1],$myPo[2],$myPo[3]);
764. //    all.append([subject, predicate, myPo[1]])


768. foreach ($obj as $o) $all[]=array($subject,$predicate, $o[1],$o[2],$o[3]);
769. //         for x in obj: all.append([subject, predicate, x])

771. }


775. $r = $this->getObjs($spo);
776. $spo=$r[0];

778. $objs=$r[1];

780. //spo, objs = getObjs(spo)
781. $subject=$spo[0];
782. $predicate=$spo[1];


785. if(!isset($spo[3])) $spo[3]=' ';
786. if(!isset($spo[4])) $spo[4]=' ';

788. $all[]=array($subject, $predicate, $spo[2],$spo[3],$spo[4]);

790. foreach ($objs as $obj) $all[]=array($subject, $predicate, $obj[1],$obj[2],$obj[3]);

792. return $all;
793. }

795. /**
796. * Makes lists of elements in list into a seperate array element.
797. * e.g. doLists(["a","b","[","c","]","d"], "[","]")=> ["a","b", ["c"], "d"]
798. * @param array $list
799. * @param string $schar
800. * @param string $echar
801. * @returns array
802. * @access private
803. ***/
804. function doLists($list, $schar, $echar) {

806. while (in_array($schar, $list)) {
807. //   while schar in list {
808. $ndict=array();
809. $nestingLevel=0;
810. $biggest=0;
811. for ($i=0;$i<count($list);$i++) {
812. if ($list[$i] == $schar) {
813. $nestingLevel += 1;
814. if (!in_array($nestingLevel, array_keys($ndict))) {
815. $ndict[$nestingLevel] = array(array($i));
816. } else {
817. $ndict[$nestingLevel][]=array($i);
818. }
819. }
820. if ($list[$i] == $echar) {
821. if (!in_array($nestingLevel, array_keys($ndict))) {
822. $ndict[$nestingLevel]=array(array($i));
823. } else {
824. $ndict[$nestingLevel][count($ndict[$nestingLevel])-1][]=$i;
825. $nestingLevel-= 1;
826. # elif type(list[i]) == type([]) {
827. #    list[i] = doLists(list[i], schar, echar)
828. }
829. }
830. }
831. foreach (array_keys($ndict) as $key)
832. if ($key > $biggest)  $biggest = $key;

834. $tol = $ndict[$biggest][0];
835. $list = $this->listify($list, $tol[0], ($tol[1]+1));
836. }
837. return $list;
838. }

840. /**
841. * Apply doLists for all different types of list.
842. * @param array
843. * @returns array
844. * @access private
845. ***/
846. function listStuff($list) {
847. # y, z = zip(['[', ']'], ['{', '}'], ['(', ')'])
848. # return map(doLists, [list, list, list], y, z).pop()
849. $list = $this->doLists($list, '[', ']');
850. $list = $this->doLists($list, '{', '}');
851. return $this->doLists($list, '(', ')');
852. }

854. /**
855. * Generates a new node id.
856. * @access private
857. * @returns string
858. ***/
859. function bnodeID() {

861. $this->bNode++;
862. return "_".BNODE_PREFIX.$this->bNode."_";
863. }

865. /**
866. * This makes bNodes out of variables like _:a etc.
867. * @access private
868. * @param array $list
869. * @returns array
870. ***/
871. function fixAnon($list) {
872. //    $map=array();
873. for($i=0;$i<count($list);$i++) {
874. $l=$list[$i];
875. if (substr($l,0,2)=="_:") {
876. if (!isset($this->bNodeMap[$l])) {
877. $a=$this->bnodeID();
878. $this->bNodeMap[$l]=$a;
879. } else $a=$this->bNodeMap[$l];
880. $list[$i]=$a;
881. }
882. }
883. return $list;
884. }

886. /**
887. * This makes [ ] lists into bnodes.
888. * @access private
889. * @param array $list
890. * @return array
891. ***/
892. function expandLists($list) {

894. for($i=0;$i<count($list);$i++) {
895. if (is_array($list[$i])) {
896. if ( $list[$i][0]=='[' ) {
897. $bnode=$this->bnodeID();
898. $prop=$list[$i];
899. $list[$i]=$bnode;
900. $list[]=$bnode;
901. $list=$this->array_concat($list, array_slice($prop,1,-1));
902. $list[]='.';
903. }elseif($list[$i][0]=='(') {

905. $rdfNil = '<'. RDF_NAMESPACE_URI . RDF_NIL .'>';
906. $rdfFirst = '<'. RDF_NAMESPACE_URI . RDF_FIRST .'>';
907. $rdfRest = '<'. RDF_NAMESPACE_URI . RDF_REST .'>';

909. // local copy of list without "(" and ")"
910. $t_list = array_slice($list[$i], 1, -1);

912. //prepare bnodes
913. $fromBnode = $this->bnodeID();
914. $toBnode = $this->bnodeID();

916. //link first bnode into graph
917. $list[$i] = $fromBnode;

919. $count = count($t_list);

921. //loop through list, convert to RDF linked list
922. for ($idx = 0; $idx < $count; $idx++){

924. // set rdf:first
925. $list[] = $fromBnode;
926. $list[] = $rdfFirst;
927. $list[] = $t_list[$idx];
928. $list[] = '.';

930. // set rdf:rest (nil or next bnode)
931. if ($idx == $count - 1) {
932. $list[] = $fromBnode;
933. $list[] = $rdfRest;
934. $list[] = $rdfNil;
935. $list[] = '.';
936. }
937. else {
938. $list[] = $fromBnode;
939. $list[] = $rdfRest;
940. $list[] = $toBnode;
941. $list[] = '.';

943. $fromBnode = $toBnode;
944. $toBnode = $this->bnodeID();
945. }
946. }
947. }
948. else {
949. die('Only [ ] and () lists are supported!');
950. }
951. }


954. }
955. return $list;
956. }

958. /**
959. * Main work-horse function. This converts a N3 string to a list of statements
960. * @param string $s
961. * @returns array
962. * @access private
963. ***/
964. function n3tolist($s) {

966. //   """Convert an N3 string into a list of triples as strings."""
967. $result = array();

969. $t = $this->filterWs($this->toke($s)); # tokenize the stream, and filter whitespace tokens

971. if ($this->debug) {
972. print "Filter WS:\n";
973. var_dump($t);
974. }
975. $r=$this->getPrefixes($t); # get the prefix directives, and add to a dict
976. $prefixes=$r[0];
977. $t=$r[1];
978. if ($this->debug) {
979. print "Prefixes:\n";
980. var_dump($prefixes);
981. print "***\n";
982. var_dump($t);
983. }
984. $t=$this->applyStuff($prefixes, $t);#apply prefixes, keywords, and string formatting
985. if ($this->debug) {
986. print "Stuff applied:\n";
987. var_dump($t);
988. }

990. $t=$this->fixAnon($t); # fix _:a anons
991. if ($this->debug) {
992. print "Fix anon:\n";
993. var_dump($t);
994. }

996. $t = $this->listStuff($t); # apply list stuff: todo
997. if ($this->debug) {
998. print "Lists done:\n";
999. var_dump($t);
1000. }
1001. $t=$this->expandLists($t);
1002. if ($this->debug) {
1003. print "Lists applied:\n";
1004. var_dump($t);
1005. }
1006. $t = $this->getStatements($t); # get all of the "statements" from the stream

1008. foreach ($t as $stat) {
1009. $stats=$this->statementize($stat);

1011. foreach ($stats as $y) {
1012. $result[]=$y;
1013. }
1014. }
1015. //   for x in [statementize(stat) for stat in t] {
1016. //      for y in x: result.append(y)
1017. return $result;
1018. }

1020. /**
1021. * Constructs a RAP RDFNode from URI/Literal/Bnode
1022. * @access private
1023. * @param string $s
1024. * @returns object RDFNode
1025. ***/
1026. function toRDFNode($s,$state) {
1027. $ins=substr($s,1,-1);
1028. if ($s{0}=="\"") {
1029. $lang=NULL;


1033. if (count($state)>3) {


1036. for ($i = 3; $i < count($state); $i++){
1037. if ($state[$i][0]=='@')$lang=substr($state[3],1);
1038. if (substr($state[$i],0,2)=='^^'){

1040. $dtype=substr($state[$i],2);
1041. if ($dtype[0]=='<') $dtype= substr($dtype,1,-1);

1043. };

1045. };
1046. };


1049. if(UNIC_RDF){
1050. $ins=$this->str2unicode_nfc($ins);
1051. }
1052. $new_Literal=new Literal($ins,$lang);
1053. if (isset($dtype)) $new_Literal->setDatatype($dtype);
1054. return  $new_Literal;
1055. };

1057. if (strstr($s,'_'.BNODE_PREFIX)) {
1058. if (($this->FixBnodes) OR (!array_search($s,$this->bNodeMap))) {
1059. return new BlankNode($ins);
1060. } else {return new BlankNode(trim(substr(array_search($s,$this->bNodeMap),2)));
1061. };
1062. }

1064. return new Resource($ins);
1065. }


1070. } //end: N3Parser

1072. ?>
```

Documentation generated on Thu, 7 Jul 2005 13:42:11 +0200 by phpDocumentor 1.3.0RC3
